# Simultaneous EEG and fNIRS recordings for semantic decoding of imagined animals and tools

**DOI:** 10.1038/s41597-025-04967-0

**Published:** 2025-04-12

**Authors:** Milan Rybář, Riccardo Poli, Ian Daly

**Affiliations:** Brain-Computer Interfaces and Neural Engineering Laboratory, School of Computer Science and Electronic Engineering, University of Essex, Colchester, CO4 3SQ United Kingdom

**Keywords:** Cognitive neuroscience, Biomedical engineering, Computational science, Neural decoding

## Abstract

Semantic neural decoding aims to identify which semantic concepts an individual focuses on at a given moment based on recordings of their brain activity. We investigated the feasibility of semantic neural decoding to develop a new type of brain-computer interface (BCI) that allows direct communication of semantic concepts, bypassing the character-by-character spelling used in current BCI systems. We provide data from our study to differentiate between two semantic categories of animals and tools during a silent naming task and three intuitive sensory-based imagery tasks using visual, auditory, and tactile perception. Participants were instructed to visualize an object (animal or tool) in their minds, imagine the sounds produced by the object, and imagine the feeling of touching the object. Simultaneous electroencephalography (EEG) and near-infrared spectroscopy (fNIRS) signals were recorded from 12 participants. Additionally, EEG signals were recorded from 7 other participants in a follow-up experiment focusing solely on the auditory imagery task. These datasets can serve as a valuable resource for researchers investigating semantic neural decoding, brain-computer interfaces, and mental imagery.

## Background & Summary

Semantic concepts are mental constructs that represent knowledge and understanding in the human mind. They play a vital role in how we perceive and process the world around us^1^. Recent advancements in cognitive neuroscience, machine learning, and the science of human language have demonstrated the possibility of semantic neural decoding. This decoding involves identifying the specific semantic concepts an individual is concentrating on or thinking about at a given moment in time by analyzing their brain activity^2,3^.

Our research aims to investigate the feasibility of applying semantic neural decoding to brain-computer interfaces (BCIs) designed for communication^4,5^. BCIs establish a direct pathway between the brain and external devices, bypassing the traditional musculoskeletal system. These systems have been explored for diverse applications, including assistive technologies for individuals with disabilities^6^ and entertainment and gaming for the general population^7^. However, current BCIs face challenges, particularly in terms of communication speed and accuracy, which remain inferior to conventional methods^8–10^.

Semantic BCIs, which leverage semantic neural decoding, could enable the direct transmission of conceptual meaning. This approach contrasts with existing state-of-the-art BCI spellers, which transmit characters one at a time, requiring the completion of entire words to convey meaning^6,11,12^. Semantic BCIs have the potential to address this limitation, offering a more intuitive mode of communication. Nonetheless, it is currently unknown whether such systems can achieve the accuracy and speed necessary for practical use.

While current studies highlight the exciting possibility of semantic neural decoding, they employ a wide range of semantic concepts, mental tasks, experimental designs, and machine learning techniques^3^. Importantly, not all of these approaches are suitable for BCI applications. A key requirement for semantic BCIs is that they must operate without relying on external cues to guide user choices.

The most promising results in semantic neural decoding have been achieved using functional magnetic resonance imaging (fMRI)^13–16^. This non-invasive, whole-brain neuroimaging technique measures brain activity by detecting changes associated with cerebral blood flow. Despite its effectiveness, fMRI has significant limitations, including high costs, lack of portability, low temporal resolution, and the restrictive environment of the scanner, which limits the range of cognitive tasks and abilities that can be studied. For these reasons, decoding semantic information from neural signals captured non-invasively at the scalp, such as through electroencephalography (EEG) or functional near-infrared spectroscopy (fNIRS), has become an attractive alternative for developing semantic BCIs. EEG measures electrical activity at the scalp surface. However, because the signals pass through multiple layers of non-neural tissue, they are highly attenuated and noisy, resulting in a lower spatial resolution of around 2 cm compared to fMRI. Nevertheless, EEG excels in temporal resolution, offering millisecond-level precision. fNIRS is a non-invasive technique that measures cortical brain activity up to approximately 2 cm in depth by detecting hemodynamic responses associated with neuronal activity, similarly to fMRI. Its key advantages are portability and lower costs compared to fMRI, making it a promising substitute^17^. Combining EEG and fNIRS recordings presents an ideal solution for semantic BCIs, as these techniques complement each other. Both are portable, cost-effective, and better suited to real-world applications compared to fMRI. EEG provides excellent temporal resolution, while fNIRS has the potential to address EEG’s poor spatial resolution. Together, they offer a synergistic approach to improving the ecological validity and practicality of semantic BCIs.

Before implementing mental tasks without external cues in BCIs, it is essential to validate these mental tasks within an experimental design where participants are cued with a specific semantic concept. This design should clearly separate the cue presentation period from the mental task period^18^. To this end, we designed an experiment to explore the feasibility of distinguishing between the semantic categories of animals and tools using four distinct mental tasks.

The first mental task, silent naming, required participants to silently name a displayed object on the screen in their minds. This task has been widely used in previous studies^19–23^, but its feasibility for semantic neural decoding using fNIRS had not yet been evaluated. We addressed this gap by testing silent naming in our experiment^24^.

Several studies^14,15,21,25–37^ have instructed participants to think about the properties or meanings of concepts or to generate mental images. Research consistently shows that perceiving objects and imagining them elicit similar brain activity patterns^38–41^. Following this approach, we focused on mental imagery tasks^38,42– 44^, where participants freely used mental imagery to think about concepts. Mental imagery is a core cognitive ability common to most people^38,45,46^, making it a natural basis for mental tasks. Building on this, we introduced three novel sensory-based imagery tasks grounded in visual, auditory, and tactile modalities. Participants were instructed to: (a) visualize the object in their minds, (b) imagine the sounds the object might produce, and (c) imagine the feeling of touching the object.

Simultaneous EEG and fNIRS data were collected from 12 participants during these four mental tasks. Additionally, a simplified version of the experiment focused solely on the auditory imagery task was conducted, with only EEG data recorded from 7 participants. We refer to the primary dataset as Dataset 1 and the simplified version as Dataset 2. We believe these datasets will prove valuable for researchers studying semantic neural decoding, BCIs, or mental imagery^38,42– 44,46,47^.

## Methods

### Participants

#### Dataset 1

Twelve right-handed native English speakers (3 males, 9 females) were recruited from the student and staff community at the University of Essex. Participants ranged in age from 20 to 57 years, with a mean age of 32.75 years and a standard deviation of 11.55 (see Table 1 for additional details). To mitigate potential variability in the neural representation of semantic concepts caused by language differences^48,49^, only native English speakers were included, a factor particularly relevant to the silent naming task. All participants had normal or corrected-to-normal vision and received £16 as compensation for their time. They all read, understood, and signed a consent form for their data to be made publicly available in anonymized form for research purposes. The study was approved by the Ethics Committee of the University of Essex on 25 October 2018. The experiment was performed in accordance with relevant guidelines and regulations. This experiment was also described in^18,24,50^.

**Table 1:** Participant information for Dataset 1.

Participant	Age	Sex	Handedness	fNIRS montage
1	26	female	right	frontal
2	32	female	right	frontal
3	57	male	right	frontal
4	47	female	right	frontal
5	23	female	right	frontal
6	21	female	right	frontal
7	29	female	right	temporal
8	50	female	right	temporal
9	27	female	right	temporal
10	33	female	right	temporal
11	28	male	right	temporal
12	20	male	right	temporal

Adapted from^50^.

#### Dataset 2

Seven right-handed individuals (5 males, 2 females) were recruited from our lab. Their ages ranged from 27 to 44 years, with a mean age of 34 years and a standard deviation of 5.98 (see Table 2 for additional details). Unlike Dataset 1, this experiment did not include the silent naming task, so native English fluency was not required. In fact, none of the participants were native English speakers. All participants had normal or corrected-to-normal vision and provided written informed consent prior to the experiment for their data to be made publicly available in anonymized form for research purposes. The research protocol was approved by the Ethics Committee of the University of Essex on 20 February 2023 (ETH2223-0805). The experiment was performed in accordance with relevant guidelines and regulations. This experiment was also described in^18^.

**Table 2:** Participant information for Dataset 2.

Participant	Age	Sex	Handedness
1	44	male	right
2	33	male	right
3	35	female	right
4	37	male	right
5	25	female	right
6	37	male	right
7	27	male	right

### Mental tasks

#### Dataset 1

Participants were shown images representing concepts from two semantic categories: animals and tools. After viewing each image, they performed four distinct mental tasks: silent naming, visual imagery, auditory imagery, and tactile imagery. The order of these mental tasks was randomized across blocks.

In the silent naming task, participants were instructed to silently name the displayed object in their minds in their mother tongue (English). In the visual imagery task, they were asked to visualize the object, focusing on their own mental representation rather than the specific image presented. For the auditory imagery task, participants imagined the sounds associated with the object. For instance, the sounds made by an animal (such as the mewing of a cat) or the sounds produced when using a tool (such as the banging of a hammer). Finally, in the tactile imagery task, participants imagined the feeling of touching the object, such as petting an animal or touching different parts of a tool.

Descriptions of these mental tasks, including the above-mentioned examples, were provided beforehand, but participants were encouraged to use the imagery strategy that felt most natural to them. During each mental task, participants were instructed to remain engaged for the full 3-second duration and to minimize physical movements, including eye movements, facial expressions, and head or body motions. To avoid biasing participants’ interpretations of the images, all images were shown to them before the experiment. The name of an image was only provided if a participant could not identify it; otherwise, they were free to rely on their own interpretation.

#### Dataset 2

The experimental design for Dataset 2 was similar to that of Dataset 1, with two key differences: (1) participants only performed the auditory imagery task, and (2) the duration of the mental task was extended to 5 seconds, compared to the 3 seconds used in Dataset 1.

### Stimuli

The study utilized a set of 18 animals and 18 tools. These semantic categories and cuing using the visual modality (images) have been extensively used in prior semantic decoding studies^3^. The selected concepts were chosen for their suitability across all mental tasks and their recognizability by a broad audience. For example, certain animals were excluded if they were deemed unsuitable for the auditory imagery task, as many participants might struggle to recall and imagine the sounds those animals produce. Images were sourced from the Internet under licenses permitting non-commercial reuse with modifications. Each image was converted to gray-scale, cropped, resized to 400 × 400 pixels, and contrast stretched. The objects were presented against a white background. Photographic images were used instead of line drawings to allow for a greater set of potential images.

The selected concepts are as follows:

**Animals**. bear, cat, cow, crab, crow, dog, donkey, duck, elephant, frog, lion, monkey, owl, pig, rooster, sheep, snake, and tiger.

**Tools**. axe, bottel-opener, broom, chain saw, computer keyboard, computer mouse, corkscrew, hammer, hand saw, hoover, kettle, knife, microwave, pen, phone, scissors, shovel, and toothbrush.

### Experimental design

#### Dataset 1

**Fig. 1:**
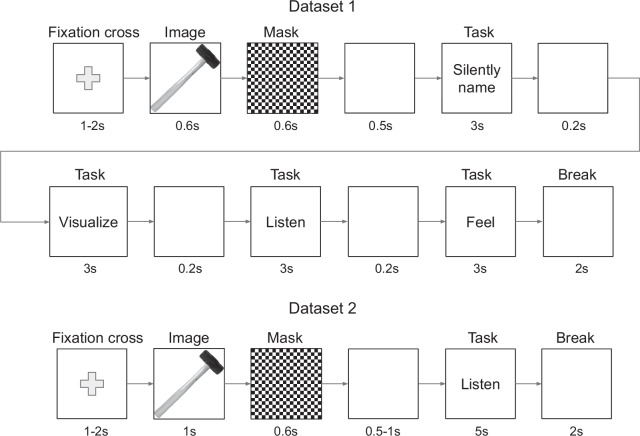
Illustration of a single concept trial in Datasets 1 and 2. In Dataset 1, the order of mental tasks was randomized across blocks. Based on^18^.

The structure of a single concept trial is shown in Fig. 1. Each concept trial started with a black fixation cross displayed on a white background for a duration of 1–2 seconds (uniformly distributed). Following this, the image of a concept was shown for 0.6 seconds. To minimize visual persistence and eliminate potential effects of perceptual-processing-related neural activity following the image presentation^51^, a checkerboard mask was displayed for another 0.6 seconds. Afterward, a blank white screen appeared for 0.5 seconds before a sequence of four mental tasks. Each mental task lasted for 3 seconds, with a blank white screen displayed for 0.2 seconds between mental tasks. The type of mental task was indicated by text on the screen throughout the mental task duration: “Silently name”, “Visualize”, “Listen”, or “Feel”. After completing the final mental task, a 2-second break followed, represented by a blank screen that gradually transitioned from white to black and back to white. Overall, a single concept trial lasted 17.3–18.3 seconds, depending on the duration of the fixation cross.

Each concept was presented five times, resulting in a total of 90 trials per category (18 concepts, 5 repetitions each). The experiment consisted of 15 blocks, each containing 12 concepts, lasting 207.6–219.6 seconds per block. Blocks were separated by breaks of at least 30 seconds, with a longer break of at least 3 minutes occurring in the middle of the experiment. The experiment began with two additional short familiarization blocks (86.5–91.5 seconds), each containing a random subset of five concepts repeated twice.

The order of concepts and mental tasks was pseudo-randomized with the following constraints: (1) no concept was repeated twice in succession, (2) all mental tasks within a block had the same order, and (3) no mental task order was repeated in the following block.

#### Dataset 2

Dataset 2 was recorded using a simplified version of the Dataset 1 experiment, focusing only on the auditory imagery task (see Fig. 1). The cue presentation time for the concept image was extended to 1 second, and the auditory imagery task duration was increased to 5 seconds. Additionally, the blank screen before the mental task was adjusted to last 0.5–1 second (uniformly distributed). Each concept trial lasted 10.1–11.6 seconds. Concepts were presented seven times, increasing the total number of trials per category to 126 (18 concepts, 7 repetitions each). The experiment consisted of 14 blocks, each containing 18 concepts, lasting 181.8–208.8 seconds per block. Breaks between blocks were the same as in the first experiment. Concept order was pseudo-randomized as in Dataset 1 to prevent consecutive repetitions of the same concept, with an additional constraint to balance semantic categories within each block.

### Data acquisition

#### Dataset 1

EEG data were recorded using a BioSemi ActiveTwo system with 64 electrodes placed according to the international 10-20 system. Two additional electrodes were positioned on the earlobes as references. Galvanic skin response (GSR) was measured using two electrodes on the left hand, and respiration was recorded using a belt placed around the waist; all signals were recorded by the same BioSemi ActiveTwo system. The sampling rate for EEG, GSR, and respiration was 2048 Hz. fNIRS data were recorded using a NIRx NIRScoutXP continuous wave imaging system equipped with 4 light detectors, 8 light emitters (sources), and low-profile fNIRS optodes. Both EEG electrodes and fNIRS optodes were placed into a NIRx NIRScap for integrated fNIRS-EEG layouts. Synchronization between EEG and fNIRS signals was achieved by sending triggers from the experimental control computer at the onset of each event. Triggers were delivered to both systems via a parallel port and an active parallel port splitter box.

The selection of brain regions for fNIRS optode placement was guided by the semantic processing network identified by Binder *et al*.^52^ Their meta-analysis of 120 functional neuroimaging studies revealed a left-lateralized network associated with semantic processing comprised of 7 regions: posterior inferior parietal lobe (angular gyrus and adjacent supramarginal gyrus), middle temporal gyrus (and posterior portions of the inferior temporal gyrus), fusiform and parahippocampal gyri, dorsomedial prefrontal cortex, inferior frontal gyrus (especially pars orbitalis), ventromedial prefrontal cortex, and posterior cingulate gyrus (and adjacent ventral precuneus). These findings are consistent with semantic decoding studies^3^. Given that fNIRS can only measure cortical activity close to the scalp, we focused on three left-lateralized regions accessible to fNIRS: the posterior inferior parietal lobe, the middle temporal gyrus, and the dorsomedial prefrontal cortex.

**Fig. 2:**
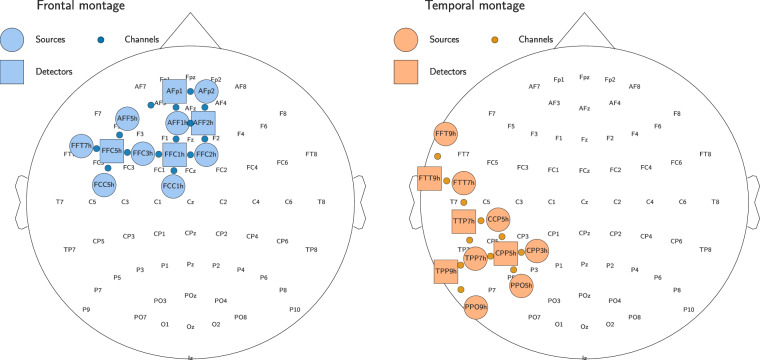
The frontal and temporal montages used for fNIRS data acquisition in Dataset 1 alongside the joint EEG system with 64 electrodes, following the international 10-20 system. The fNIRS sources (depicted as circles) and detectors (depicted as squares) were positioned according to the 10-5 system, forming channels (depicted as small circles) located between the sources and detectors.

Two distinct fNIRS montages were created due to limitations in the number of channels available with our equipment (see Fig. 2). The montages were designed using the fOLD toolbox^53^, which optimizes optode placement based on regions of interest. The first montage targeted the left temporal lobe and the posterior inferior parietal lobe. Regions of interest for the fOLD toolbox were the left-lateralized inferior parietal lobe, angular gyrus, middle and inferior temporal gyrus. Optodes with the lowest specificity to target regions were excluded to meet hardware constraints. The montage included 4 detectors and 7 sources, producing 11 channels with a sampling rate of 8.92 Hz. The second montage focused on the left frontal cortex. Regions of interest for the fOLD toolbox were Brodmann areas 9 and 46. The montage included 4 detectors and 8 sources, producing 14 channels with a sampling rate of 7.81 Hz. The inter-optode separation for both montages was approximately 3 cm. The frontal montage was used for the first six participants, while the temporal montage was used for the remaining six participants (see Table 1).

#### Dataset 2

In Dataset 2, only EEG data were recorded. EEG acquisition followed the same protocol as in Dataset 1, with additional electrooculography (EOG) recordings to monitor eye movements. Two electrodes were placed above and below the right eye to capture the vertical oculogram, while two more electrodes were placed near the canthus of each eye to record the horizontal oculogram. Unlike in Dataset 1, galvanic skin response was not measured. Instead, two electrodes were placed on the left and right wrists for additional physiological measurements (e.g., heart rate variability).

### Order of tasks in Dataset 1

**Fig. 3:**
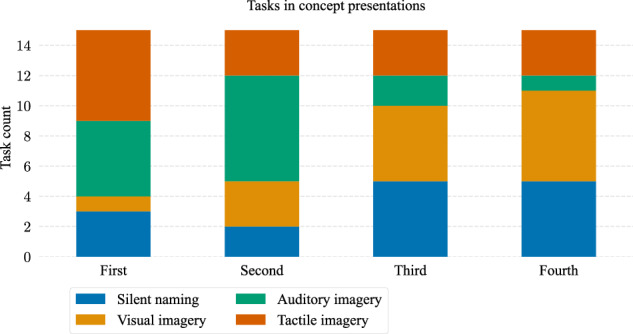
The frequency of mental task appearances as the first, second, third, and fourth tasks in the shared order of mental tasks for participants 2 to 12 in Dataset 1.

During data analysis, a minor scripting error was identified in the experimental program used for Dataset 1. Specifically, the random number generator responsible for shuffling the order of concepts and mental tasks was initialized with the same seed for all participants except participant 1. As a result, participants 2–12 were presented with identical sequences of mental tasks and concepts. Figure 3 illustrates the frequency distribution of mental tasks appearing in the first, second, third, and fourth positions within blocks. The distribution is non-uniform, with a skew toward presenting the auditory imagery and tactile imagery tasks more frequently as the first task, and the auditory imagery task more often as the second task. Note that if the ease of semantic decoding is influenced by the position of the mental task within the sequence of four tasks, the randomization seed initialization error could adversely impact the results. For example, if the first mental task presented, irrespective of its type, consistently provides the most distinguishing features for differentiating between semantic categories, this could lead to a bias. In particular, the tactile and auditory imagery tasks, which were presented more frequently than the other tasks (especially compared to the visual imagery task), may benefit from this bias. As a result, they may contribute more samples to the training process within the classification pipeline, potentially leading to higher classification performance for these mental tasks.

## Data Records

Datasets can be accessed from the OpenNeuro platform via 10.18112/openneuro.ds004514.v1.1.2^54^ for Dataset 1 and 10.18112/openneuro.ds004517.v1.0.2^55^ for Dataset 2. All datasets have been structured following the Brain Imaging Data Structure (BIDS) standard^56^, which supports EEG^57^ and fNIRS^58^ data. In compliance with this standard, files are first organized by participant and then categorized into modality-specific folders. EEG data are stored in BioSemi .bdf files, with corresponding event annotations and channel information saved in .tsv files. fNIRS data are stored in the Shared Near Infrared Spectroscopy Format (SNIRF) as .snirf files, with corresponding event annotations, channel information, and optode locations also stored in .tsv files. At the root level, participant information is stored in a .tsv file, accompanied by a .json file that describes each column. All stimulus images are located in the stimuli folder, with references to these images included in the stim_file column of the event .tsv files. The original data were converted into BIDS format with the help of the MNE-BIDS package^59^.

## Technical Validation

The quality of the EEG and fNIRS signals was ensured through multiple procedures conducted both during and after data acquisition. EEG electrode quality was assessed during setup using voltage offsets displayed in Biosemi ActiView software and the quality of the EEG time series. The voltage offsets were monitored to ensure they remained within the acceptable range (below 20). Similarly, the quality of each fNIRS channel was evaluated using the calibration procedure in NIRx NIRStar software, which provided initial feedback on signal quality for each channel. It was not always possible to obtain good-quality fNIRS channels due to variations in participants’ hair size, thickness, and density^60,61^. The datasets contain raw EEG and fNIRS data including bad channels. This allows users the flexibility to apply their preferred preprocessing methods.

**Fig. 4:**
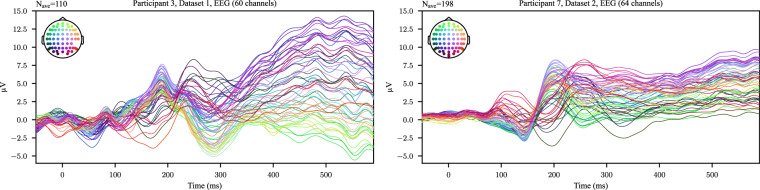
Examples of average EEG ERPs with respect to the image presentation for a single participant from Dataset 1 and 2. *N*_*ave*_ represents the number of trials over which the channel data were averaged.

**Fig. 5:**
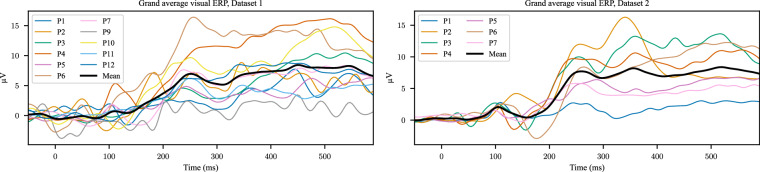
The grand average visual EEG ERP with respect to the image presentation for each participant. The black bold line indicates the average across participants.

The EEG data were analyzed in our previous manuscripts^18,50^. EEG signals were referenced to the mean of electrodes placed on the left and right earlobes. Manual inspection was performed to identify electrodes exhibiting abnormal signal behavior. This inspection was based on visual assessment of raw EEG traces for artifacts such as excessive noise, drifts, or flat signals. Participant 8 in Dataset 1 was excluded from further EEG analysis due to overall poor signal quality and the presence of artifacts in the majority of concept trials. Eye-blink artifacts were identified and suppressed using component analysis^62,63^. Full details can be found in our previous manuscripts^18,50^. Figure 4 shows example average event-related potentials (ERPs) for a single participant with respect to image presentation from each dataset, demonstrating a clear neural response to the stimulus. The shown EEG signals were filtered between 0.1 and 30 Hz. Additionally, Fig. 5 shows the grand average visual ERP for each participant, showing consistent patterns of brain activity related the image presentation.

**Fig. 6:**
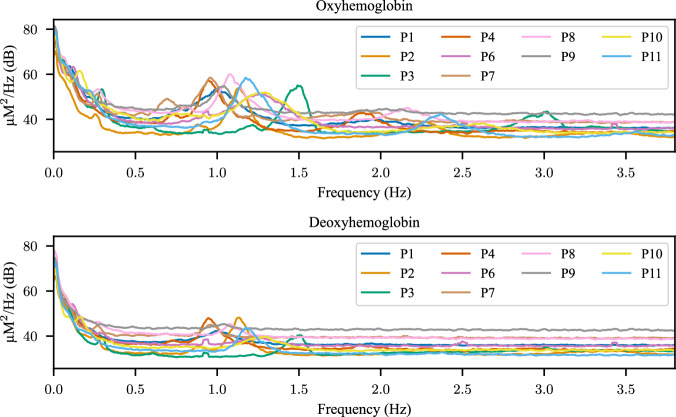
The average fNIRS power spectrum for each participant in Dataset 1.

**Fig. 7:**
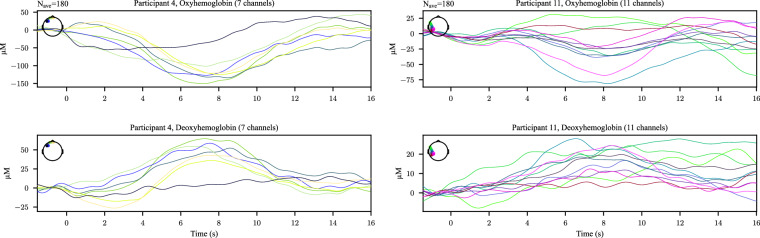
Examples of fNIRS ERPs with respect to the image presentation for two participants in Dataset 1, the first one with the frontal montage and the second one with with the temporal montage. *N*_*ave*_ represents the number of trials over which the channel data were averaged.

The fNIRS data were analyzed in our previous manuscripts^24,50^. Bad channels were identified using the scalp coupling index (SCI)^64^. The most prominent artifact in the fNIRS signal is the cardiac cycle, visible at approximately 1 Hz. This artifact reflects intracranial physiological activity and serves as a marker of good contact between the optical probe and the scalp^65,66^. Bad channels identified by the SCI generally matched those flagged during the initial calibration process. Two participants (participants 5 and 12) were excluded from further fNIRS analysis because they had the majority of channels removed. Figure 6 shows the average power spectrum for each participant, where the cardiac cycle is clearly visible and varies across individuals. Motion artifacts caused by relative motion between the optical fibers and the scalp were corrected using a wavelet transform method^67–69^ implemented in the Homer2 software package^70^. Motion-corrected signals at wavelengths 785 nm and 830 nm were converted into changes in concentration of oxygenated and deoxygenated hemoglobin using the modified Beer-Lambert law^71^. The resulting hemoglobin concentration signals were filtered between 0.01 and 0.7 Hz to remove low-frequency drift and the cardiac cycle. Full details can be found in our previous manuscripts^24,50^. Figure 7 shows example fNIRS event-related responses for a single participant of each montage, demonstrating clear changes in oxygenated and deoxygenated hemoglobin concentrations with respect to image presentation. These responses follow the expected relationship between changes in oxygenated and deoxygenated hemoglobin.

## Usage Notes

The data are stored in the BIDS format^56–58^, ensuring easy accessibility using standard software and analysis packages. The provided datasets include raw EEG signals recorded with the BioSemi system. These signals are not referenced. It is recommended to apply referencing prior to analysis, such as using the mean of the ear references.

One notable limitation of these datasets is the relatively small number of participants for the fNIRS data in Dataset 1 (6 participants per montage) and for the EEG data in Dataset 2 (7 participants). While the data collected from these participants provided valuable insights and allowed for the investigation of the research questions, the limited sample size may impact the generalizability of the findings. A small sample size can reduce the statistical power of the analysis and may increase the likelihood of Type I or Type II errors. Additionally, the variability inherent in individual differences among participants may not be fully captured, potentially affecting the robustness of the conclusions. Future research should aim to include a larger and more diverse cohort of participants to validate and extend the findings. This would help ensure that the results are more representative of the broader population and enhance the reliability of the conclusions.

## Data Availability

We have prepared example scripts to demonstrate how to load the EEG and fNIRS data into Python using MNE^72^ and MNE-BIDS^59^ packages. These scripts are located in the code directory.
